# A telomere-to-telomere gap-free reference genome assembly of avocado provides useful resources for identifying genes related to fatty acid biosynthesis and disease resistance

**DOI:** 10.1093/hr/uhae119

**Published:** 2024-04-22

**Authors:** Tianyu Yang, Yifan Cai, Tianping Huang, Danni Yang, Xingyu Yang, Xin Yin, Chengjun Zhang, Yunqiang Yang, Yongping Yang

**Affiliations:** CAS Key Laboratory of Tropical Plant Resources and Sustainable Use, Xishuangbanna Tropical Botanical Garden, Chinese Academy of Sciences, Kunming, Yunnan 650223, China; Germplasm Bank of Wild Species, Yunnan Key Laboratory of Crop Wild Relatives Omics, Kunming Institute of Botany, Chinese Academy of Sciences, Kunming, Yunnan 650201, China; School of Life Sciences, Yunnan University, Kunming, Yunnan 650091, China; Kunming College of Life Science, University of Chinese Academy of Sciences, Beijing 100049, China; CAS Key Laboratory of Tropical Plant Resources and Sustainable Use, Xishuangbanna Tropical Botanical Garden, Chinese Academy of Sciences, Kunming, Yunnan 650223, China; CAS Key Laboratory of Tropical Plant Resources and Sustainable Use, Xishuangbanna Tropical Botanical Garden, Chinese Academy of Sciences, Kunming, Yunnan 650223, China; Center of Gardening & Horticulture, Xishuangbanna Tropical Botanical Garden, Chinese Academy of Sciences, Menglun, Mengla, Yunnan 666303, China; CAS Key Laboratory of Tropical Plant Resources and Sustainable Use, Xishuangbanna Tropical Botanical Garden, Chinese Academy of Sciences, Kunming, Yunnan 650223, China; CAS Key Laboratory of Tropical Plant Resources and Sustainable Use, Xishuangbanna Tropical Botanical Garden, Chinese Academy of Sciences, Kunming, Yunnan 650223, China; Kunming College of Life Science, University of Chinese Academy of Sciences, Beijing 100049, China; CAS Key Laboratory of Tropical Plant Resources and Sustainable Use, Xishuangbanna Tropical Botanical Garden, Chinese Academy of Sciences, Kunming, Yunnan 650223, China; Germplasm Bank of Wild Species, Yunnan Key Laboratory of Crop Wild Relatives Omics, Kunming Institute of Botany, Chinese Academy of Sciences, Kunming, Yunnan 650201, China; CAS Key Laboratory of Tropical Plant Resources and Sustainable Use, Xishuangbanna Tropical Botanical Garden, Chinese Academy of Sciences, Kunming, Yunnan 650223, China; Germplasm Bank of Wild Species, Yunnan Key Laboratory of Crop Wild Relatives Omics, Kunming Institute of Botany, Chinese Academy of Sciences, Kunming, Yunnan 650201, China; Institute of Tibetan Plateau Research at Kunming, Kunming Institute of Botany, Chinese Academy of Sciences, Kunming, Yunnan 650201, China; CAS Key Laboratory of Tropical Plant Resources and Sustainable Use, Xishuangbanna Tropical Botanical Garden, Chinese Academy of Sciences, Kunming, Yunnan 650223, China; Germplasm Bank of Wild Species, Yunnan Key Laboratory of Crop Wild Relatives Omics, Kunming Institute of Botany, Chinese Academy of Sciences, Kunming, Yunnan 650201, China; Institute of Tibetan Plateau Research at Kunming, Kunming Institute of Botany, Chinese Academy of Sciences, Kunming, Yunnan 650201, China

## Abstract

Avocado (*Persea americana* Mill.) is an economically valuable plant because of the high fatty acid content and unique flavor of its fruits. Its fatty acid content, especially the relatively high unsaturated fatty acid content, provides significant health benefits. We herein present a telomere-to-telomere gapless genome assembly (841.6 Mb) of West Indian avocado. The genome contains 40 629 predicted protein-coding genes. Repeat sequences account for 57.9% of the genome. Notably, all telomeres, centromeres, and a nucleolar organizing region are included in this genome. Fragments from these three regions were observed via fluorescence *in situ* hybridization. We identified 376 potential disease resistance-related nucleotide-binding leucine-rich repeat genes. These genes, which are typically clustered on chromosomes, may be derived from gene duplication events. Five *NLR* genes (*Pa11g0262*, *Pa02g4855*, *Pa07g3139*, *Pa07g0383*, and *Pa02g3196*) were highly expressed in leaves, stems, and fruits, indicating they may be involved in avocado disease responses in multiple tissues. We also identified 128 genes associated with fatty acid biosynthesis and analyzed their expression patterns in leaves, stems, and fruits. *Pa02g0113*, which encodes one of 11 stearoyl-acyl carrier protein desaturases mediating C18 unsaturated fatty acid synthesis, was more highly expressed in the leaves than in the stems and fruits. These findings provide valuable insights that enhance our understanding of fatty acid biosynthesis in avocado.

## Introduction

Avocado (*Persea americana* Mill.) is a tropical evergreen woody plant species originating from Central America. Its fruits are rich in nutritious, health-promoting, disease-preventing metabolites and have a creamy texture and a unique aroma because of a high fatty acid content, especially unsaturated fatty acid [1]. Thus, avocado has been consumed for over 5000 years and represents a globally economically valuable crop [2, 3]. Over 8 million metric tons of avocado were produced worldwide in 2021 [4]. Several tropical countries, such as Mexico, Colombia, Peru, Indonesia, Dominican Republic, and Kenya, are major avocado producers, with an output exceeding 6.8 million metric tons in 2021 [4]. However, avocado production is beset by challenges. In Kenya, a major avocado exporter, more than 60% of avocado fruits do not meet international market standards because of low quality and damages due to anthracnose disease [5]. Wilt disease caused by *Phytophthora cinnamomi* has resulted in yield losses of ~40%–90% in Colombia and 20%–25% in California, USA, where 5% of the avocado-planting area is affected [6]. In addition, a disease induced by nectriaceous fungi has been detected in various regions (e.g. Australia, Chile, Colombia, and Italy), leading to considerable economic losses in the avocado industry [7–11].

There has been interest in the potential utility of genes encoding nucleotide-binding leucine-rich repeat receptor (NLR) proteins, which reportedly contribute to disease resistance. On the basis of a transcriptome analysis, Pérez-Torres et al. [12] determined that the expression levels of four unigenes (*UN003976*, *UN001791*, *UN003288*, and *UN003220*) encoding coiled-coil-type NLR proteins increased during the early stages of an infection by *Fusarium kuroshium*. Furthermore, there may be tissue-specific NLR network responses to specific pathogens in plants [12]. Therefore, identifying and functionally annotating *NLR* genes in avocado is critical for exploring their roles in immune responses to diseases.

As an economic crop with a rich cultivation history, avocado has been studied in terms of its substantial fatty acid content. The initiation of fatty acid biosynthesis involves acetyl coenzyme A (acetyl-CoA) and biochemical reactions in plastids that produce the 16:0-acyl carrier protein (ACP). Subsequently, 16:0-ACP is modified by numerous enzymatic reactions, with the resulting long-chain acyl-CoA recatalyzed and stored in the acyl-CoA pool within the endoplasmic reticulum. Concurrently, C18:0 and C18:1 are bound to malonyl-CoA through sequential reactions, similar to fatty acid synthesis in plastids, yielding desaturated long-chain fatty acids, which are stored in the phosphatidylcholine (PC) pool for subsequent processes. The products stored in the acyl-CoA Pool and PC pool are channeled into the Kennedy pathway, leading to the formation of triacylglycerols (TAGs) [13]. In most plants, the enzymatic reactions associated with C18 unsaturated fatty acid biosynthesis have been well established. The major unsaturated fatty acids in plants are oleic (18:1), linoleic (18:2), and α-linolenic (18:3) acids (i.e. C18 species) [14]. The formation of unsaturated fatty acids is mainly regulated by three specialized fatty acid desaturases (FADs) (i.e. acyl-lipid, acyl-ACP, and acyl-CoA desaturases) [15]. There has been relatively little research on the expression patterns and functions of the genes encoding enzymes involved in unsaturated fatty acid formation in avocado.

With the development of third-generation sequencing technologies, which produce much longer and more accurate reads than previous sequencing technologies, several plant telomere-to-telomere (T2T) genome assemblies have been generated for various species, including *Arabidopsis thaliana* [16, 17], *Oryza sativa* [18, 19], *Brassica rapa* [20], *Actinidia chinensis* [21, 22], *Rhododendron molle* [23], and *Rhodomyrtus tomentosa* [24]. Gapless T2T genomes are useful for studying centromeres [17, 18, 20], which are dynamic, rapidly evolving chromosomal regions critical for maintaining chromosomal integrity and genetic information fidelity during cell division [25, 26]. Earlier research showed that centromeric regions are usually highly methylated and contain repetitive satellite DNA sequences (satellites) and transposable elements (TEs), including long terminal repeats (LTRs) [17, 18, 20]. These highly repetitive, complex sequences can make it difficult to analyze plant centromere structures and functions [26–28]. Research on plant centromeres has been limited to model plants and crop species, such as *A. thaliana* with a 178-bp CEN178 (formerly known as CEN180) [29], rice with a 155-bp CentO [30], *Zea mays* with a 156-bp CentC [31], and *Triticum aestivum* with a 566-bp CentT566 [32]. There has been a lack of research on centromeres in avocado.

Several studies have generated valuable genomic data relevant to avocado research [12, 33–38], including two Hass avocado genome assemblies with 12 chromosomes [33, 34]. However, considering avocado is an economically valuable tropical plant species, its genome must be more comprehensively characterized. In this study, we generated a gapless T2T genome assembly for West Indian avocado by integrating multiple sequencing technologies, which includes all the telomeres, centromeres, and a nucleolar organizing region (NOR). These regions were validated via fluorescence *in situ* hybridization (FISH). Additionally, we analyzed the expression of *NLR* genes and genes associated with fatty acid biosynthesis in various avocado tissues. The T2T genome assembly described herein may form the basis of future research on disease resistance and fatty acid biosynthesis in avocado.

## Results

### Gap-free avocado genome assembly

**Figure 1:**
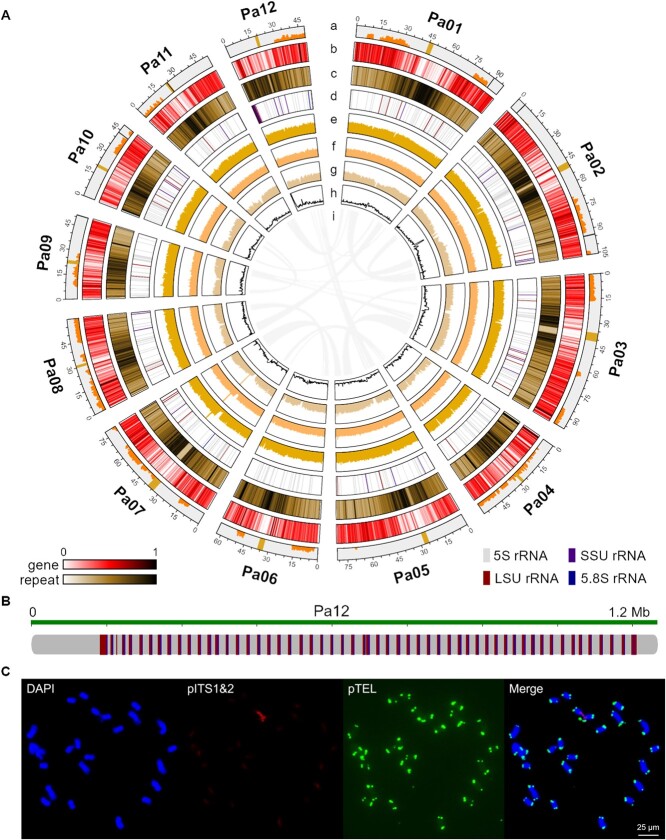
Landscape of the telomere-to-telomere gap-free avocado genome assembly**.** (A) Genome landscape Circos plot. (a) Chromosomes with gap-filling locations in black, estimated centromeres in gold, and heterozygous site density in orange red bars; (b) gene density; (c) repeat density; (d) rRNA location; (e) HiFi read depth; (f) ONT ultra-long read depth; (g) NGS read depth; (h) GC content; (i) intra-genomic collinearity. Densities and depths were calculated in 500-kb windows with 250-kb steps along chromosomes. (B) 45S rDNA array on Pa12. (C) Fluorescence signals of FISH probes pITS1&2 (red) and pTEL (green) indicate the locations of NOR and telomeres on avocado mitotic metaphase chromosomes.

Multiple sequencing technologies were used to sequence the genome of a West Indian avocado plant collected from Xishuangbanna Tropical Botanical Garden, China. A preliminary genome survey, which was performed using 51.9 Gb paired-end reads generated by whole-genome next-generation sequencing (NGS) revealed the genome size (864 Mb) and heterozygosity rate (0.637%) (Fig. S1). On the basis of this genome size, several sequencing platforms were used to obtain the following data: 70.9 Gb (82.1×) of PacBio HiFi reads with an N50 of 17.7 kb, 39.3 Gb (45.5×) of ONT ultra-long reads with an N50 of 100.3 kb, and 89.8 Gb (104.0×) of Pore-C reads (Table S1). The HiFi reads and ONT ultra-long reads were used along with hifiasm [39] to construct a highly accurate preliminary assembly with an N50 of 63.6 Mb (Table S2). After discarding organelle fragments and redundant sequences, contigs were clustered, ordered, and oriented using wf-pore-c [40] and juicebox [41] pipelines with manual validation (Fig. S2). A total of 18 contigs with significant contact signals were anchored onto 12 chromosomes, seven of which were gap-free, and six gaps were added to scaffold 11 contigs into five chromosomes (Table S3). Chromosome identification numbers and orientations were refined according to a published avocado genome [34] (Pa01–Pa12) (Table S3). Assemblies generated by several assemblers were used to fill gaps (Table S2). Contigs that could bridge any gap were used as input data of quarTeT [42] for automated gap filling, and then the filled gaps were manually validated. Thus a gap-free genome assembly was obtained. Several genomic regions were found to have low HiFi and ONT read coverage depth (Fig. 1A). To ensure a correct assembly, sequences in these regions were inspected and compared among assemblies generated by different assemblers (Table S2). All these regions were either fixed (gap-filling method) or verified that they could be assembled using hifiasm with no additional gaps. The low coverage depth may be related to sequence repeatability and complexity. Telomeres were fixed by aligning and jointing candidate ONT ultra-long reads to the chromosome ends lacking telomeres. After completing all correction and polishing procedures, the final avocado genome assembly comprised 841.6 Mb and consisted of 12 gap-free chromosomes with an N50 of 78.8 Mb and 24 telomeres (Table S4).

### Genome annotation

Repeat sequences (repeats) in the avocado assembly were identified using the Extensive de novo TE Annotator (EDTA) pipeline [43]. Additionally, a repeat library was obtained after TEs were classified. According to the EDTA analysis, repeats accounted for 57.9% of the assembly (Table S5). The most common repeats were LTR/Copia (7.3%) and LTR/Gypsy (22.1%) retrotransposons (Table S5). The repeat library was used to softmask the assembly. Gene models were predicted using the softmasked assembly and BRAKER3 [44], which combined the results of transcriptome-based, homologous protein-based, and *ab initio* predictions. We obtained 40 629 protein-coding gene models. The genes were distributed on both chromosomal arms in a symmetrical pattern, whereas the repeats were concentrated in relatively central regions (Fig. 1A). The proteins encoded by these genes included homologs of 32 645 and 23 485 proteins in the non-redundant (NR) and Swiss-Prot databases, respectively. InterProScan [45, 46] and eggNOG-mapper [47, 48] assigned Pfam, Gene Ontology (GO), and KEGG Orthology (KO) terms to 24 877, 13 977, and 13 786 proteins, respectively (Table S6). Furthermore, we identified heterozygous sequences (4 118 925 bp) at 3 158 398 sites by remapping HiFi reads to the genome (Fig. 1A). Most of these sequences were in intergenic and intronic regions. There were 98 128 heterozygous sites in exonic regions, including 53 628 nonsynonymous single nucleotide variants that altered 1850 transcription start or termination sites.

Noncoding RNAs were predicted by infernal cmscan [49] and Rfam [50] databases. The prediction resulted in 458 transfer RNAs, 398 small nucleolar RNAs, 177 microRNAs, and 3576 5S ribosomal RNAs (Table S7). The NOR detected on Pa12 contained dozens of 45S rDNA units, which comprised a set of small subunit rRNA, internal transcribed spacer1 (ITS1), 5.8S rRNA, ITS2, and large subunit rRNA arranged head to tail (Fig. 1B). NOR is important for ribosome and nucleolus formation during interphase [51]. It may also be responsible for the high GC content at the end of Pa12 (Fig. 1A)**.** The *A. thaliana*-type telomeric repeats (TTTAGGG/CCCTAAA) were used to identify telomeres in this avocado assembly. The ends of all chromosomes contained a telomeric region ranging from 4683 bp to 27 191 bp in length (Table S8). To validate the authenticity of NOR and telomeres revealed by the assembly, we designed FISH probes (pITS1, pITS2, and pTEL) on the basis of the NOR and telomere sequences (Table S9). According to the red and green fluorescence signals, there was a pair of NORs among 12 pairs of chromosomes. Moreover, all chromosomes had telomeric regions at each end (Fig. 1C), which was in accordance with the results of the bioinformatics analysis.

### Quality assessment and validation

We used multiple methods to evaluate assembly quality. The overall mapping rates of HiFi reads, ONT ultra-long reads, and NGS reads were 99.55%, 99.91%, and 97.86%, respectively. Coverage breadths of all chromosomes exceeded 99.9%, and coverage depth was generally uniform among chromosomes (Fig. 1A; Table S10). Moreover, the overall alignment rates of RNA-seq reads generated from leaves, stems, and fruits were greater than 99.1% (Table S11). By elucidating the correct order and orientation of sequences, the Pore-C contact heatmap verified the continuity of the assembly (Fig. S2). Merqury [52] was used to calculate the base-level quality values of the genome on the basis of HiFi reads (overall value of 56.23) (Table S12). The LTR Assembly Index (LAI) [53] score calculated using intact LTR-RTs was 15.99, which reaches the reference standard. Finally, a Benchmarking Universal Single-Copy Orthologs (BUSCO) [54] analysis (in protein mode) captured 1604 of 1614 conserved genes (99.4%) in embryophyta_odb10 (Table S13). These results reflect the high continuity, accuracy, and integrity of this avocado genome assembly.

### Avocado centromere characterization

**Figure 2:**
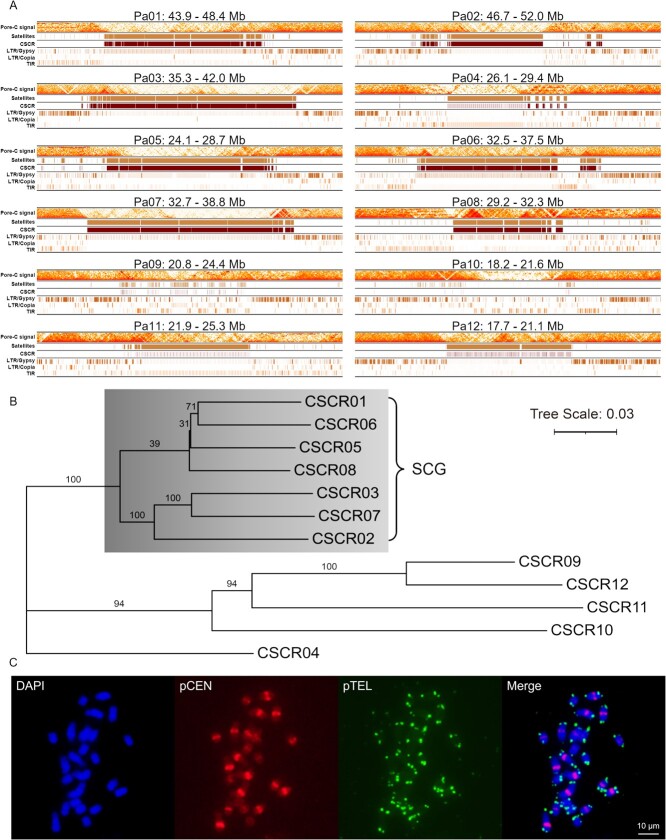
Centromeric architecture in avocado. (A) Tracks showing Pore-C contact signal near-absent regions, putative satellite locations, CSCR locations, LTR/Gypsy locations, LTR/Copia locations, and TIR locations. CSCR, chromosome-specific centromeric repeat; LTR, long terminal repeat; and TIR, terminal inverted repeat. Pore-C contact signals were calculated in 15-kb bins. Putative satellite and TE locations were determined using RepeatMasker, whereas CSCR locations were determined according to LASTZ alignments. (B) Neighbor-joining tree showing phylogenetic relationships of CSCRs. The gray clade comprises seven highly homologous CSCRs, which were designated as the Seven CSCRs Group (SCG). CSCRs were aligned using the MAFFT einsi algorithm. The neighbor-joining tree was constructed using TreeBeST. (C) Fluorescence signals of FISH probes pCEN (red) and pTEL (green) indicate the locations of centromeres and telomeres on avocado mitotic metaphase chromosomes.

Iterative identification and clustering methods were used to estimate centromere locations on chromosomes (Fig. 1A). A total of 12 chromosome-specific centromeric repeats (CSCR) in the corresponding chromosome centromeres were identified and designated as CSCR01 to CSCR12 (Table S14). Most CSCRs were longer than 1000 bp, which exceeds the length of published centromeric monomers. Seven CSCRs (CSCR01, CSCR02, CSCR03, CSCR05, CSCR06, CSCR07, and CSCR08) had similar sequences, with identity and coverage exceeding 83.0% and 98.7%, respectively (Table S15; Fig. S3), and always appeared on the corresponding centromeres in a head-to-tail orientation (Fig. 2A). These CSCRs formed the Seven CSCRs Group (SCG) (Fig. 2B). CSCR04, CSCR11, and CSCR12 on non-SCG chromosomes were arranged in intervals, whereas CSCR09 and CSCR10 were relatively rare on the corresponding chromosomes. These CSCRs were somewhat similar to SCG according to the LASTZ and MAFFT alignments (Table S15; Fig. S3). The Vsearch [55] clustering results indicated that CSCR01 (alternatively called PaCEN1016) can serve as a representative avocado centromeric monomer. The Pore-C signal near-absent regions and CSCR locations were used for the determination of centromere borders on each chromosome (Table S16). Multiple locations in these complex regions had low HiFi and ONT read coverages, especially the long centromeric regions of Pa03 and Pa07 (Fig. 1A). To validate the authenticity of these regions and CSCRs, we designed a FISH probe (pCEN) on the basis of the consensus CSCR sequences (Table S9, Table S14, and Fig. S3). Red fluorescence signals confirmed the existence of these CSCRs (Fig. 2C).

The 1 Mb regions flanking centromeres included CSCRs together with satellites and TEs. There was considerable overlap between LTR/Gypsy and SCG-rich regions, whereas non-SCG centromeres included multiple types of TEs (Fig. 2A). The alignments of these CSCRs to the sequences in the repeat library generated by the repeat annotation pipeline revealed the substantial similarity between these CSCRs and a number of TEs (Table S15). Notably, CSCR01 contained the sequences of three TEs (Fig. S4; Table S15). Thus, these CSCRs may have been derived from TEs. These findings indicate that TE insertions may have largely shaped the centromere structure in avocado.

### Structural variation analysis

**Figure 3:**
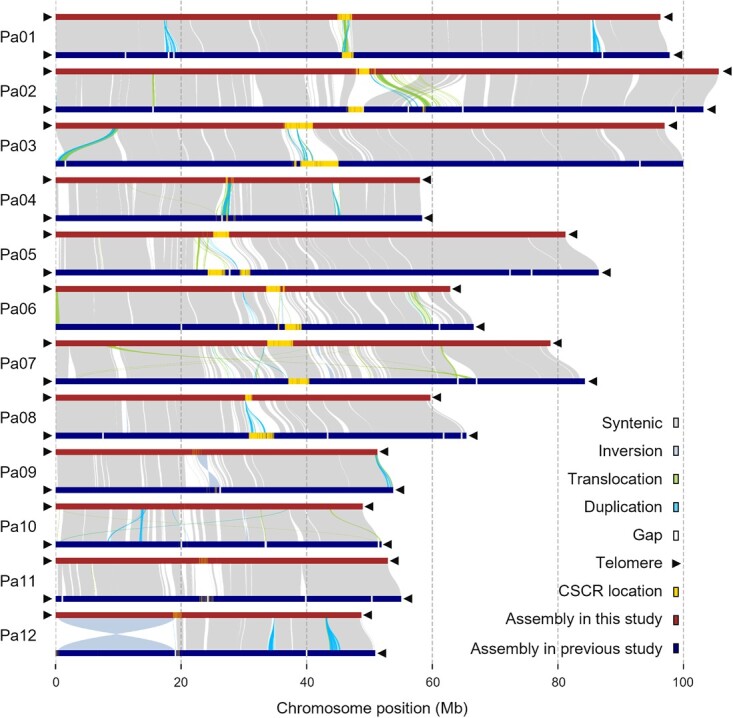
Structural variations among avocado assemblies. Collinear syntenic blocks, inversions, translocations, and duplications are shown between homologous chromosomes. Chromosome-specific centromeric repeats and gaps on chromosomes are marked in yellow and white, respectively. Black triangles indicate telomeres.

To screen for differences between the previously assembled Hass avocado genome and our West Indian avocado genome, we analyzed their structural variations (Fig. 3; Fig. S5). Large-scale structural rearrangements were mainly detected near complex centromeric regions. Examples include the translocation on Pa02 and inversion on Pa12 (Fig. S6). A total of 582 485 insertions/deletions (InDels) were identified, of which 7668 insertions and 7685 deletions were longer than 50 bp (Table S17). Gene-based annotation detected 5700 InDels in the exonic regions of 4373 genes, many of which encode protein kinases, disease resistance-related proteins, transcription factors, and cytochrome P450, in the West Indian avocado genome (Table S18).

### Exploring *NLR* genes in avocado

**Figure 4:**
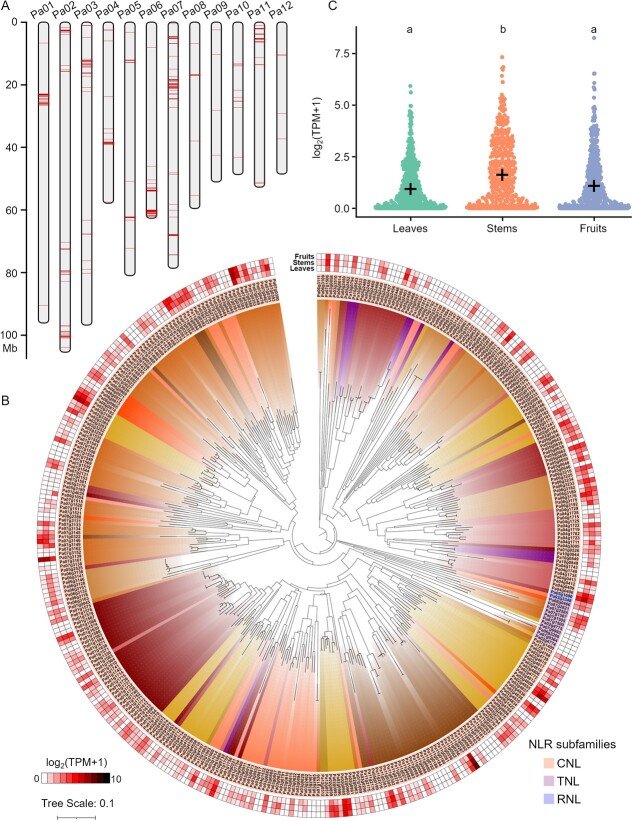
Phylogenetic and transcriptome analyses of *NLR* genes in avocado. (A) Clustered distribution of *NLR* genes on chromosomes, with gene locations marked in red. (B) Neighbor-joining tree of avocado NLR proteins. The heatmap behind the tree shows the relative expression levels of the corresponding genes in leaves, stems, and fruits. The branches from identical chromosomes are marked by the same color. The different colors of gene ID represent CNL, TNL, and RNL subfamilies of *NLR* genes. **C** Jitter plot showing TPM values of *NLR* genes in leaves, stems, and fruits. The significance of any differences was assessed by an analysis of variance followed by Tukey’s HSD test.

To analyze the potential disease resistance-related *NLR* genes in avocado, we identified 376 and 230 *NLR* genes in the West Indian and Hass assemblies, respectively, using NLR-Annotator and InterProScan. These *NLR* genes were rarely located in Hass assembly gap regions (Fig. S7). The diversity in the number of *NLR* genes may be due to varietal differences. On the basis of domain architectures, 376 *NLR* genes could be classified into three subfamilies, including Coiled-Coil NB-ARC Leucine-rich-repeat (CNL), Toll/interleukin-1 receptor NB-ARC Leucine-rich-repeat (TNL), and Resistance to Powdery Mildew Locus 8 NB-ARC Leucine-rich-repeat (RNL), among which the subfamily CNL contains 363 members, accounted for 96.54% of the total (Fig. 4; Table S19). GO term and KEGG pathway enrichment analyses were conducted to functionally annotate the *NLR* genes. Notably, 80 genes were annotated with the ‘response to biotic stimulus’ (GO:0009607) GO term (Table S20), whereas 154 genes were associated with the ‘plant-pathogen interaction’ (KEGG: ko04626) pathway (Table S21).

**Figure 5:**
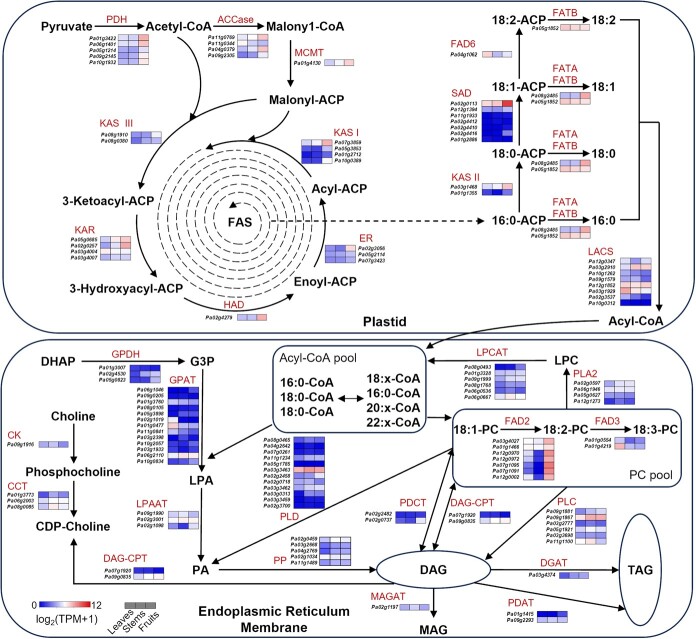
Analysis of genes involved in the fatty acid biosynthesis pathway. Heatmaps present relative expression levels in leaves, stems, and fruits. TPM values were calculated as the mean value of three replicates. Gene expression levels are normalized and represented as log_2_(TPM + 1). Blue and red represent low and high expression levels, respectively. Abbreviations: PDH, pyruvate dehydrogenase; ACCase, acetyl-CoA carboxylase; MCMT, malonyl-CoA:ACP malonyltransferase; KAS III, ketoacyl-ACP synthase III; KAR, ketoacyl-ACP reductase; HAD, hydroxyacyl-ACP dehydrase; ER, enoyl-ACP reductase; KAS I, ketoacyl-ACP synthase I; KAS II, ketoacyl-ACP synthase II; SAD, stearoyl-ACP desaturase; FAD6, fatty acid desaturase 6; FATA, acyl-ACP thioesterase A; FATB, acyl-ACP thioesterase B; LACS, long-chain acyl-CoA synthetase; GPDH, glycerol-phosphate dehydrogenase; GPAT, glycerol-3-phosphate acyltransferase; LPAAT, 2-lysophosphatidic acid acyltransferase; PP, phosphatidate phosphatase; PLD, phospholipase D; PDCT, phosphatidylcholine diacylglycerol cholinephosphotransferase; DAG-CPT, diacylglycerol choline phosphotransferase; PLC, phospholipase C; DGAT, acyl-CoA:diacylglycerol acyltransferase; MAGAT, monoacylglycerol acyltransferase; PDAT, phospholipid:diacylglycerol acyltransferase; FAD2, fatty acid desaturase 2; FAD3, fatty acid desaturase 3; PLA2, phospholipase A2; LPCAT, 2-lysophosphatidylcholine acyltransferase; ACP, acyl carrier protein; G3P, glycerol 3-phosphate; LPA, lysophosphatidic acid; PA, phosphatidic acid; DAG, diacylglycerol; TAG, triacylglycerol; PC, phosphatidylcholine; LPC, 2-lysophosphatidylcholine.

The *NLR* genes were generally distributed in clusters throughout the genome (Fig. 4A). A neighbor-joining phylogenetic tree was constructed using the protein sequences encoded by these *NLR* genes (Fig. 4B). Numerous *NLR* genes with close physical proximity on chromosome were clustered together, reflecting their close phylogenetic relationships (Fig. 4A, B). DupGen_finder results indicated that these genes may have originated from gene duplication events (e.g. whole genome duplication, tandem duplication, proximal duplication, transposed duplication, and dispersed duplication) (Table S22). Most of these *NLR* genes were derived from dispersed or proximal duplication events (Table S23). In some duplicated gene pairs, one gene lacked NLR domains, possibly because of functional differentiation or loss during evolution; these genes were not considered as *NLR* genes (Table S24).

We also analyzed *NLR* gene expression profiles in avocado leaves, stems, and fruits. Interestingly, the overall relative expression levels of these *NLR* genes were higher in the stems than in the leaves and fruits (Fig. 4C; Table S25), but some genes were highly expressed in all three tissues (e.g. *Pa11g0262*, *Pa02g4855*, *Pa07g3139*, *Pa07g0383*, and *Pa02g3196*). Accordingly, these genes may be involved in disease responses in all avocado plant tissues. Some genes, especially *Pa02g2791* and *Pa09g1054*, were expressed specifically in the stems and leaves. Additionally, our analysis of the expression profiles of *NLR* paralogous gene pairs revealed differences in their expression patterns among tissues. For example, *Pa02g4855* was expressed at very high levels, whereas its paralog *Pa02g4837* was expressed at almost undetectable levels in all three tissues. These results underscore the potential functional diversity among *NLR* genes and reflect the functional divergence between paralogous gene pairs.

### Expression analysis of fatty acid biosynthesis pathway genes

The fatty acid content is a key trait influencing the nutrient composition and quality of avocado fruits. Fatty acid biosynthesis involves biochemical processes that occur in two distinct stages: *de novo* fatty acid synthesis within plastids and TAG formation in the endoplasmic reticulum. By sequence alignments and functional annotation, we identified 128 genes associated with fatty acid biosynthesis (Fig. 5; Table S26), of which 48 and 80 genes were associated with *de novo* synthesis in plastids and TAG formation in the endoplasmic reticulum, respectively. Genes encoding three classes of enzymes, pyruvate dehydrogenase (PDH), acetyl-CoA carboxylase (ACCase), and malonyl-CoA:ACP malonyltransferase (MCMT), which are important for malonyl-ACP synthesis within plastids, were most highly expressed in fruits (Fig. 5; Table S26). In addition, the expression levels of the fatty acid synthesis-related genes *Pa08g1910*, which belongs to the ketoacyl-ACP synthase (KAS) III family, as well as *Pa02g0257* (ketoacyl-ACP reductase, KAR), *Pa02g4279* (hydroxyacyl-ACP dehydrase, HAD), *Pa02g3056* (enoyl-ACP reductase, ER), and *Pa05g3853* (KAS I) were approximately 10-times higher in the fruits than in the leaves or stems. Furthermore, *Pa02g0113*, which encodes one of the 11 stearoyl-ACP desaturases (SADs) that primarily catalyze C18 unsaturated fatty acid synthesis, was more highly expressed in the leaves than in the stems and fruits. During the TAG formation stage, FAD2 plays a crucial role affecting unsaturated fatty acid synthesis, with *Pa07g1095*, *Pa07g1091*, and *Pa12g0002* expressed specifically in fruits. Our results suggest the genes that were expressed at high levels or exclusively in the fruits may influence the fatty acid composition and content in avocado.

## Discussion

Avocado is an economically valuable plant because its fruits are a rich source of nutrients and have a unique flavor [56]. Previously published avocado genome assemblies were incomplete because of technology-related limitations [33, 34]. The generation of a high-quality genome assembly is necessary for avocado research. In this study, we used a combination of sequencing technologies to obtain a T2T gap-free genome assembly of avocado (Fig. 1A) and newly detected an NOR on Pa12 (Fig. 1A, B and Fig. 3). A total of 40 629 protein-coding genes and 4879 noncoding RNAs were predicted (Fig. 1A**,**  Table S6, and Table S7). Using various methods, we verified the high quality of the genome assembly and protein set.

The T2T genome resources necessary for *in silico* centromeric research are currently limited to model plants and crops [17, 18, 20, 57], with relatively little available information regarding avocado centromeres. In this study, we clarified the structural characteristics of avocado centromeres. Although CSCR sequences in the same chromosome are generally conserved and CSCR01 (i.e. PaCEN1016) may be a representative avocado centromere repeat (Fig. 2A), we also detected considerable variations among centromeres (Fig. 2B). This is in accordance with the results of earlier research on the centromeres of other species, including CEN178 in *A. thaliana* and CEN137 in the *Saccharum* complex [17, 29, 58, 59]. These CENs have another feature in common with CSCRs in SCG: they are arranged in a head-to-tail manner on chromosomes [17, 58, 59], whereas centromeric monomers in kiwifruit are arranged in regular intervals [21]. Compared with previously identified centromeric repeats in model plants and crops (up to several hundred base pairs in length) [21, 29–32], avocado CSCRs are much longer (>1000 bp) and their sequences differ considerably from the sequences of published centromeric repeats. In addition, centromeres on Pa04, Pa09, and Pa10 contain many TEs, especially LTR/Gypsy retrotransposons (Fig. 2A). Similar results were also reported for other plant species, including *B. rapa* and the *Saccharum* complex [20, 58], indicating that LTRs may have substantially modulated the centromeric architecture during evolution.

Many functionally validated disease resistance-related genes belong to the *NLR* gene family, which includes several subfamilies that differ regarding their structural domains [60, 61]. We identified 376 *NLR* genes in this avocado genome assembly, which distributed in clusters that may be coordinately regulated (Fig. 4A), thereby enabling avocado to rapidly perceive and respond to pathogen attacks [62, 63]. Our data indicated *Pa11g0262*, *Pa07g3139*, and *Pa07g0383* were most abundantly transcribed in the leaves, stems, and fruits (Table S25). *Pa11g0262* is partially homologous to *AT5G46510*, which is a disease resistance-related gene expressed during different developmental stages of *A. thaliana* [64]. *Pa07g3139* and *Pa07g0383* are homologous to *AT3G50950* and *AT3G07040*, respectively (Fig. S8), both of which encode a canonical NLR protein required for recognizing the phytopathogenic bacterium *Pseudomonas syringae* [25]. Another study determined the *NLR* unigene *UN001791* is responsive to an infection by *F. kuroshium* [12]; this unigene is highly similar to *Pa02g4855*. These results suggest that these *NLR* genes may be relevant to future research on the molecular mechanisms underlying responses to diseases in avocado.

The fatty acids in avocado fruits contain a high proportion of unsaturated fatty acids [65], which influence avocado quality. During fatty acid biosynthesis, ACCase catalyzes the committed and rate-limiting step of *de novo* fatty acid synthesis in plastids. In *Brassica napus*, the inhibition of ACCase activity leads to decreased fatty acid synthesis [66]. In the examined avocado fruits, three ACCase genes, *Pa06g1401*, *Pa09g2145*, and *Pa10g1932*, were expressed at high levels, suggesting they may affect fatty acid synthesis. Earlier research showed fatty acid compositions influence the physicochemical properties, nutritive value, and industrial uses of plant oils [67]. The formation of unsaturated fatty acids is mainly controlled by specialized fatty acid-modifying enzymes [68]. By inserting the first double bond into 18:0, SAD is a major determinant of the homeostasis between unsaturated and saturated fatty acids. In the *A. thaliana ssi2/fab2* mutant, the loss of SAD leads to the considerable accumulation of stearic acid (C18:0) and low C18:1 level [69]. Notably, in avocado fruits, one *SAD* gene (*Pa02g0113*) was expressed at significantly higher levels than the other *SAD* genes. The expression patterns of these genes provide valuable insights regarding fatty acid biosynthesis in avocado.

## Materials and methods

### Plant materials

Leaves, stems, and fruits were collected from a young and healthy West Indian avocado tree in the Xishuangbanna Tropical Botanical Garden, Yunnan province, China (101.2768 E, 21.9201 N). Samples were immediately frozen in liquid nitrogen and stored at −80°C for the subsequent whole-genome sequencing analysis and construction of the Pore-C library.

### Library construction and sequencing

Genomic DNA (gDNA) was extracted from leaves by CTAB method. After determining gDNA quality and quantity, ~8 μg size-selected (>50 kb) gDNA fragments were used for ONT ultra-long sequencing, which was completed on an Oxford Nanopore PromethION instrument. For HiFi sequencing, Pacific Biosciences SMRTbell target-size libraries were constructed according to the manufacturer’s standard protocol. Approximately 8 μg gDNA was used to construct libraries, which were screened regarding size and then sequenced on a PacBio Sequel II instrument. For paired-end sequencing, libraries were constructed according to the MGIEasy Universal DNA Library Prep Kit v1.0 protocol. For Pore-C sequencing, fresh leaves were immersed in 2% (v/v) fresh formaldehyde for DNA cross-linking, after which the Pore-C library was prepared by digesting the DNA using DpnII. For transcriptome sequencing, total RNA was extracted from leaves, stems, and fruits using TRIzol reagent. The RNA fragments with a poly-A tail were enriched and used as the template for cDNA synthesis, after which the cDNA ends were repaired, an A-tail was added, and an adapter was ligated according to the library construction protocol. High-quality NGS and transcriptome libraries were sequenced on the DNBSEQ-T7RS platform, whereas the high-quality Pore-C library was sequenced on the Oxford Nanopore PromethION instrument.

### Genome assembly and gap filling

Paired-end sequencing reads were filtered and cleaned using fastp v0.23.2 [70] (−l 140 -n 0). For the genome survey, a *K*-mer (*k* = 21) analysis was performed using Meryl v1.4 (https://github.com/marbl/meryl) and GenomeScope2 [71] (−p 2 -k 21) along with NGS clean reads. Hifiasm v0.19.5-r587 [39], Verkko v1.4 [72], NextDenovo v2.5.0 [73], and HiCanu v2.2 [74] were used to assemble the preliminary genome (Table S2). Organelle fragments were identified by aligning the assembly with TAIR10 *A. thaliana* chloroplast and mitochondrial sequences using LASTZ v1.04.22 (https://github.com/lastz/lastz). Purge_dups v1.2.5 [75] was used to remove redundant contigs. Wf-pore-c [40] was used to detect valid Pore-C signals. The valid Pore-C contact pairs file was converted to the hic format using juicebox_scripts (https://github.com/phasegenomics) and then imported into juicebox v1.11.08 [41] for clustering, ordering, and orienting. 3D-DNA v210623 [76] was used to generate the draft assembly on the basis of the review.assembly file from juicebox. NextDenovo v2.5.0 [73] (read_cutoff = 1 k; seed_cutoff = 76 246; genome_size = 864 m) was used to assemble the ONT ultra-long reads. NextPolish v1.4.1 [77] (task = 661 212) was used to polish the NextDenovo contigs according to both HiFi and NGS reads. ONT ultra-long reads and HiFi reads were assembled by Verkko v1.4 [72]. The NextDenovo and Verkko contigs were aligned to the draft assembly using minimap2 v2.24-r1122 [78] (−x asm5) to extract gap-bridging contigs, which were then used by quarTeT [42] to fill gaps.

### Correction and polishing procedures

To detect potential misassembled regions, we mapped ONT ultra-long reads, HiFi reads, and clean NGS reads to the assembly using minimap2 and Bowtie2 (−very-sensitive) to obtain coverage depth statistics. Read depths were calculated using SAMtools v1.18 [79] bedcov in 200-kb windows (−Q 10). Contigs generated by NextDenovo and Verkko were used to correct low-depth regions via the gap-filling method. NextPolish2 v0.2.0 [80] was used to polish the assembly with HiFi reads and NGS reads according to the author-suggested procedure. A Perl script was used to detect *A. thaliana*-type telomeric repeats (5′-TTTAGGG-3′ and 5'-CCCTAAA-3′) on chromosomes and in ONT ultra-long reads to screen for chromosome ends lacking telomeres and reads useful for fixing telomeres, respectively. The candidate reads were aligned to the chromosomes lacking telomeres using minimap2. The telomeric sequence on the longest mapped read was connected to the chromosome end.

### Genome annotation

EDTA v2.1.0 [43] and RepeatModeler v2.0.2 (http://www.repeatmasker.org) were used for the *de novo* identification of repeats and the construction of the repeat library. TEsorter v1.4.6 [81] (−db rexdb-plant) was used to further classify the TEs. Satellites were predicted using TAREAN v0.3.8.1–466 [82] and random 15× NGS reads as well as the galaxy online server (https://repeatexplorer-elixir.cerit-sc.cz/galaxy). The repeat library was used by RepeatMasker v4.1.2-p1 (http://www.repeatmasker.org) (−s) to softmask the assembly before predicting gene models. BRAKER v3.0.3 [44, 83] was used for the transcriptome-based, homologous protein-based, and *ab initio* predictions, which were filtered using TSEBRA v1.1.1 [84]. The annotation results were further filtered and formatted using MAKER v3.01.04 [85], gffread v0.12.7 [86], and GenomeTools v1.6.2 [87] for importing into Generic Feature Format version 3 (GFF3). Proteins were aligned to the sequences in the NR and Swiss-Prot databases using diamond v2.0.15.153 blastp (−e 1e-5 −top 1) [88]. InterProScan v5.64–96.0 [45, 46] and the eggNOG [47, 48] online server (http://eggnog-mapper.embl.de/) were used to functionally annotate proteins and assign Pfam, GO, and KO accessions to proteins. Heterozygous sequences were identified using Clair3 v0.1-r12 [89] and bcftools v1.18 [79] (filter -i GT = "het"), with HiFi reads as the input, and annotated by Annovar v2020-06-07 [90] according to a gene-based method. Cmscan in infernal v1.1.4 [49] and the Rfam database [50] were used to predict non-coding RNAs. RectChr v1.36 (https://github.com/hewm2008/RectChr) was used to visualize the 45S rDNAs in NOR.

### Probe and chromosome preparation for fluorescence *in situ* hybridization

The oligo-probes representing 45S rDNA and centromere sequences (Table S9) were designed on the basis of ITS1, ITS2, and CSCR sequences in the T2T avocado genome assembly generated in this study. The *A. thaliana*-type telomeric sequence was used to detect telomeres in avocado (Table S9). These probes were synthesized by Sangon Biotech Co., Ltd. (Shanghai, China).

The newly grown root tips of avocado seedlings exhibiting developmental consistency were carefully removed and promptly immersed in a solution containing 0.002 mol/L 8-hydroxyquinoline for 3–4 h. The subsequent preparation of mitotic metaphase chromosomes and the FISH analysis were conducted according to a slightly modified established procedure [91]. The chromosomes were counterstained with 4,6-diamidino-2-phenylindole (Vector Laboratories, Inc., Burlingame, USA) and examined using an Olympus BX-53 microscope equipped with a Photometric SenSys Olympus DP80 CCD camera (Olympus Corporation, Japan). The captured images were processed using Olympus cellSens Standard 4.1.1 software (Olympus Corporation).

### Quality assessments

Raw HiFi reads and ONT ultra-long reads were aligned to the final assembly using minimap2, whereas clean NGS reads were aligned using Bowtie2 [92]. Transcriptome reads were aligned to the assembly using HISAT2 v2.2.1 [93] (—very-sensitive). The format was converted and the overall mapping rates, coverage breadth, and coverage depth statistics were calculated using the SAMtools [79] commands sort, flagstat, coverage, and bedcov, respectively. Gene and repeat densities were calculated using bedtools v2.30.0 [94] makewindows and intersect, whereas the GC content was calculated using bedtools nuc. A genome landscape Circos plot was produced with TBtools v2.008 [95]. Base quality values of the raw assembly were calculated using Merqury v1.3 [52] and raw HiFi reads. LAI in LTR_retriever v2.9.0 [96] was used to calculate LAI scores. BUSCO v5.4.7 [54] was used to evaluate the completeness of the assembly and protein set according to the embryophyta_odb10 dataset.

### Centromere and structural variation characterization

A strategy involving iterative identification and clustering was used to detect centromeric repeats in the assembly. RepeatMasker (−s) was used to locate satellites. Satellite locations and Pore-C signal near-absent positions were considered together to estimate candidate centromere locations on each chromosome. High-frequency tandem repeat sequences identified in candidate centromere regions by TRF v4.09.1 [97] (2 7 7 80 10 502 000 -h) were retained for the genome-wide LASTZ alignment. Vsearch v2.22.1 [55] (—clusterout_sort —clusterout_id —fasta_width 0 —id 0.6 —cluster_size) was used to cluster the LASTZ hits on each chromosome, which resulted in 12 CSCRs. If coverage and identity percentages were both over 80% in an alignment hit of LASTZ, the hit was considered to be accurate. PyGenomeTracks v3.8 [98] was used to visualize the features on chromosome tracks. Structural variations were identified with minimap2 and Syri v1.6.3 [99] and annotated by Annovar v2020-06-07 [90] using a gene-based method. Plotsr v1.1.3 [100] was used to visualize structural variations.

### Gene identification and transcriptome analysis

The predicted full-length coding sequences in avocado were used by NLR-Annotator v2.1b [101] to identify NLR domains. In accordance with the accepted definition [102], genes containing at least one NB-ARC (Pfam accession PF00931), TIR (PF01582), or RPW8 (PF05659) domain were considered as *NLR* genes [102]. We combined the NLR-Annotator and InterProScan Pfam annotations to obtain *NLR* genes. GO enrichment and KEGG pathway enrichment analyses were performed using TBtools [95]. Relative expression levels were recorded as transcripts per million (TPM) values, which were calculated using RSEM v1.3.3 [103]. The clean RNA-seq reads were mapped to the assembly using STAR v2.7.10a [104] for TPM calculation. Relative *NLR* expression level differences among tissues were evaluated by an analysis of variance followed by Tukey’s HSD correction. NLR proteins were aligned using the MAFFT v7.520 [105] einsi algorithm. A neighbor-joining tree was constructed using TreeBeST v1.9.2 [106], with 1000 bootstrap iterations (nj -b 1000 -W). The sequences of *A. thaliana* proteins involved in the fatty acid biosynthesis pathway were obtained from ARALIP (http://aralip.plantbiology.msu.edu/pathways/pathways) [13] to serve as queries for the BLASTP search (−evalue 1e-5) of the protein set generated in this study. The Pfam and SMART (http://smart.emblheidelberg.de/) databases were screened to detect candidate proteins with conserved domains. Finally, all candidates were used to search the GenBank NR database.

## Acknowledgements

We thank all the members of the laboratory for their technical and analysis assistance. We thank Liwen Bianji (Edanz) (www.liwenbianji.cn/ac) for editing the English text of a draft of this manuscript. This research was supported by Yunling Scholar Project (to Yongping Yang), the Major Science and Technology Projects (202202AE090016), Yunnan Revitalization Talents Support Plan (to Yunqiang Yang), the Digitalization, development and application of biotic resource (202002AA100007), the Postdoctoral Research Funding Projects of Yunnan Province (to Xin Yin), the National Natural Science Foundation of China (32100315, 31601999, 41771123, 31590820, and 31590823), the West Light Foundation of the Chinese Academy of Sciences (to Yunqiang Yang), and the 13th Five-year Informatization Plan of Chinese Academy of Sciences, Grant No. XXH13506. The funders had no role in the design of the study and collection, analysis, and interpretation of data and in writing the manuscript.

## Author contributions

YQY and YPY designed the research. TYY, YFC, XYY, DNY, and XY analyzed the data. TPH, CJZ, YWD, YQY, and YPY contributed reagents/materials/analysis tools. TYY, YFC, and YQY wrote and reviewed the paper.

## Data availability statement

The raw sequencing data, including ONT Ultra-long reads, PacBio HiFi reads, NGS reads, Pore-C reads, and RNA-seq reads, assembly, and annotation data are accessible in Science Data Bank (https://doi.org/10.57760/sciencedb.07602).

## Conflict of interests

The authors declare no conflict of interest.

## Supplementary information

Supplementary data is available at Horticulture Research online.

## Supplementary Material

Web_Material_uhae119
