# Supplementary material for: A telomere-to-telomere gap-free reference genome assembly of avocado provides useful resources for identifying genes related to fatty acid biosynthesis and disease resistance
Source: Hortic Res. 2024 Apr 22;11(7):uhae119. doi: 10.1093/hr/uhae119 (PMC11220182; doi:10.1093/hr/uhae119)
Supplement: Web_Material_uhae119 [file web_material_uhae119.zip › Supplementary Figures.docx]

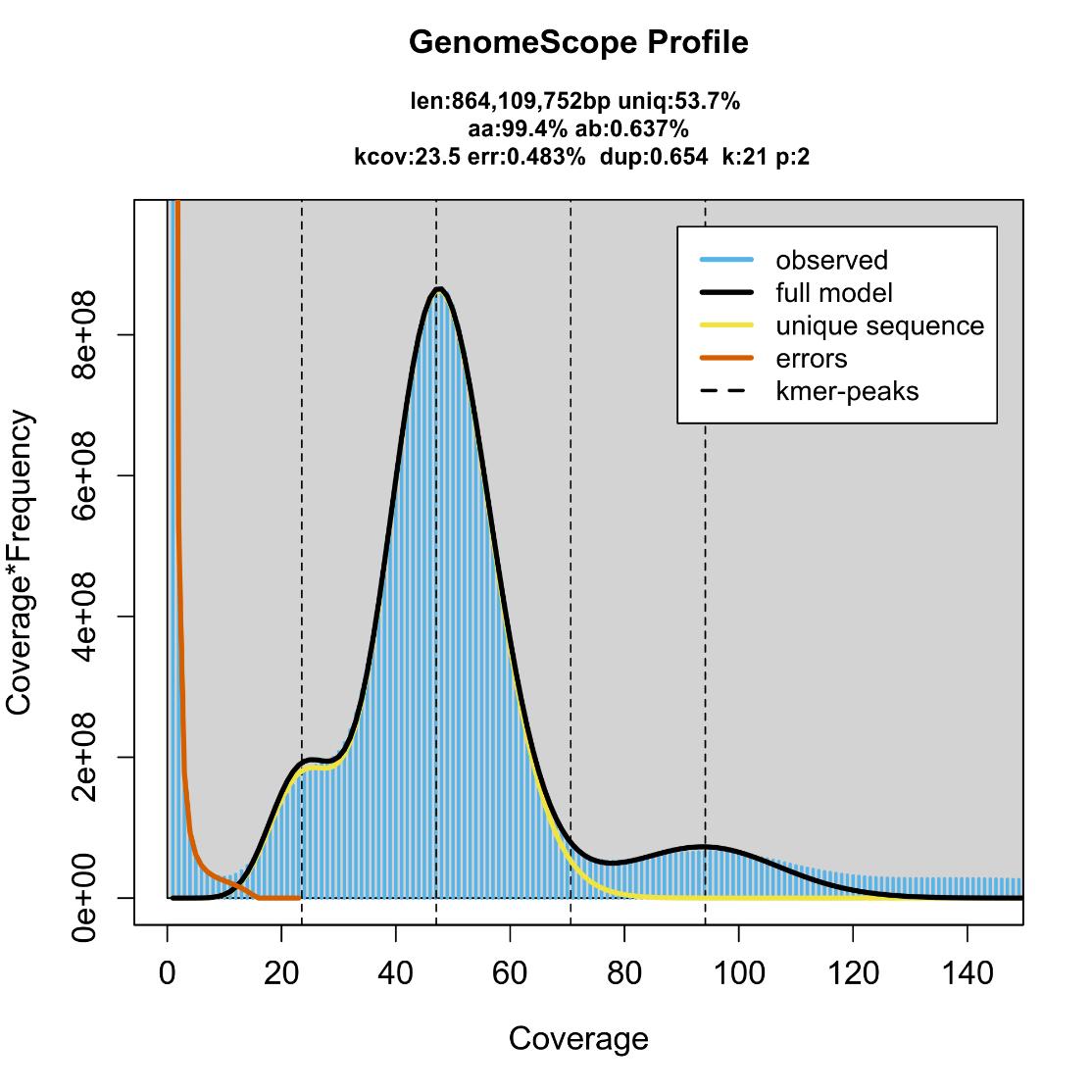
Figure S1 Genome survey results from GenomeScope2. *K*-mer (*K* = 21) density distribution showed the genome size was estimated to be 864 Mb with a heterozygosity rate of 0.637%.


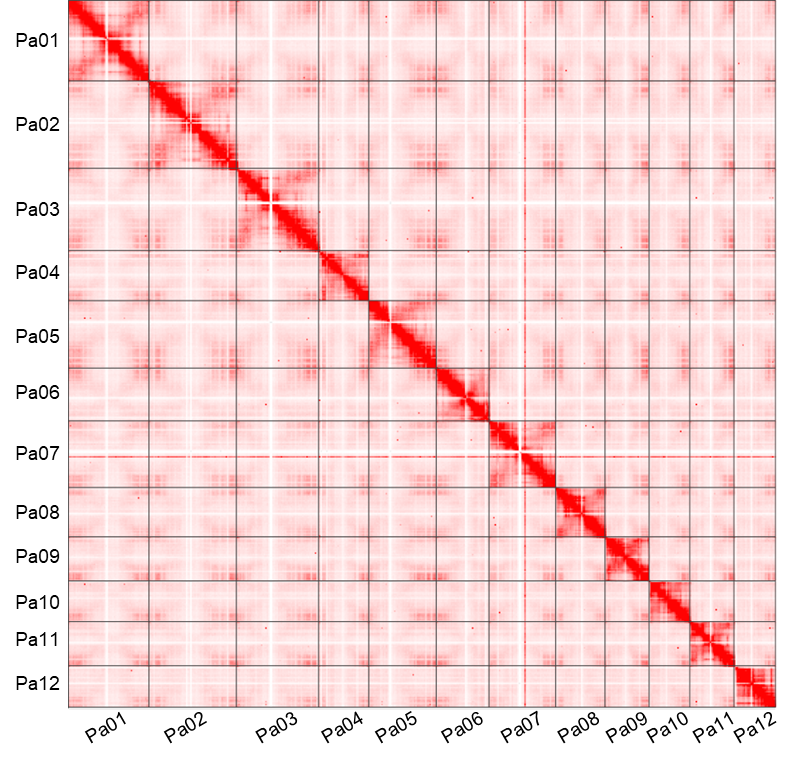
Figure S2. Whole-genome Porec-C contact heatmap with 200-kb bin.

Figure S3 Alignments among 12 CSCRs by MAFFT.


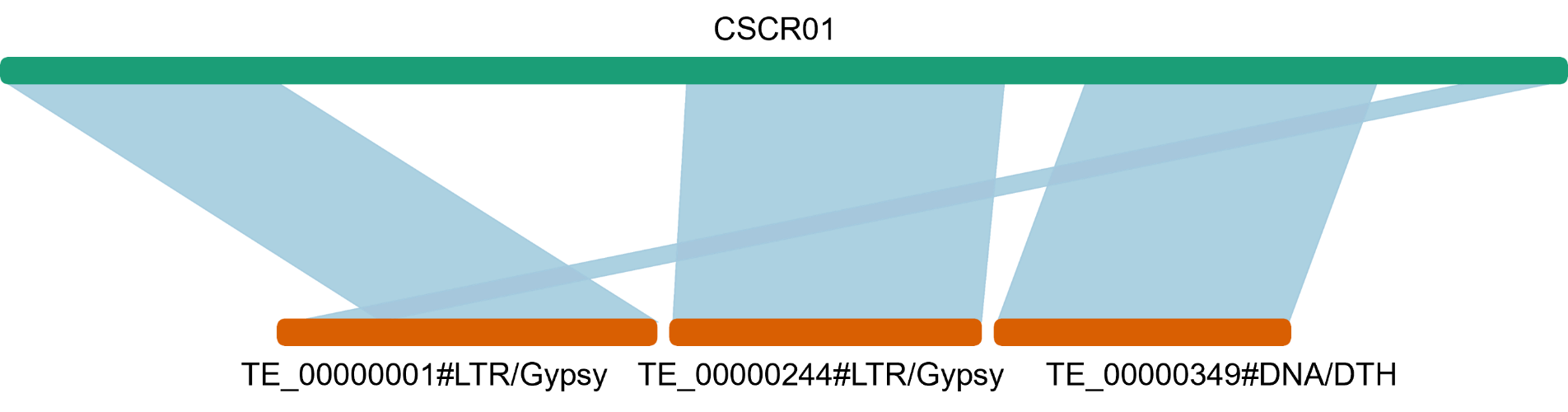
Figure S4 Collinearity between CSCR01 and three TEs. LASTZ and minimap2 alignments revealed CSCR01 sequence has high similarity with TE_00000001#LTR/Gypsy, TE_00000244#LTR/Gypsy and TE_00000349#DNA/DTH.


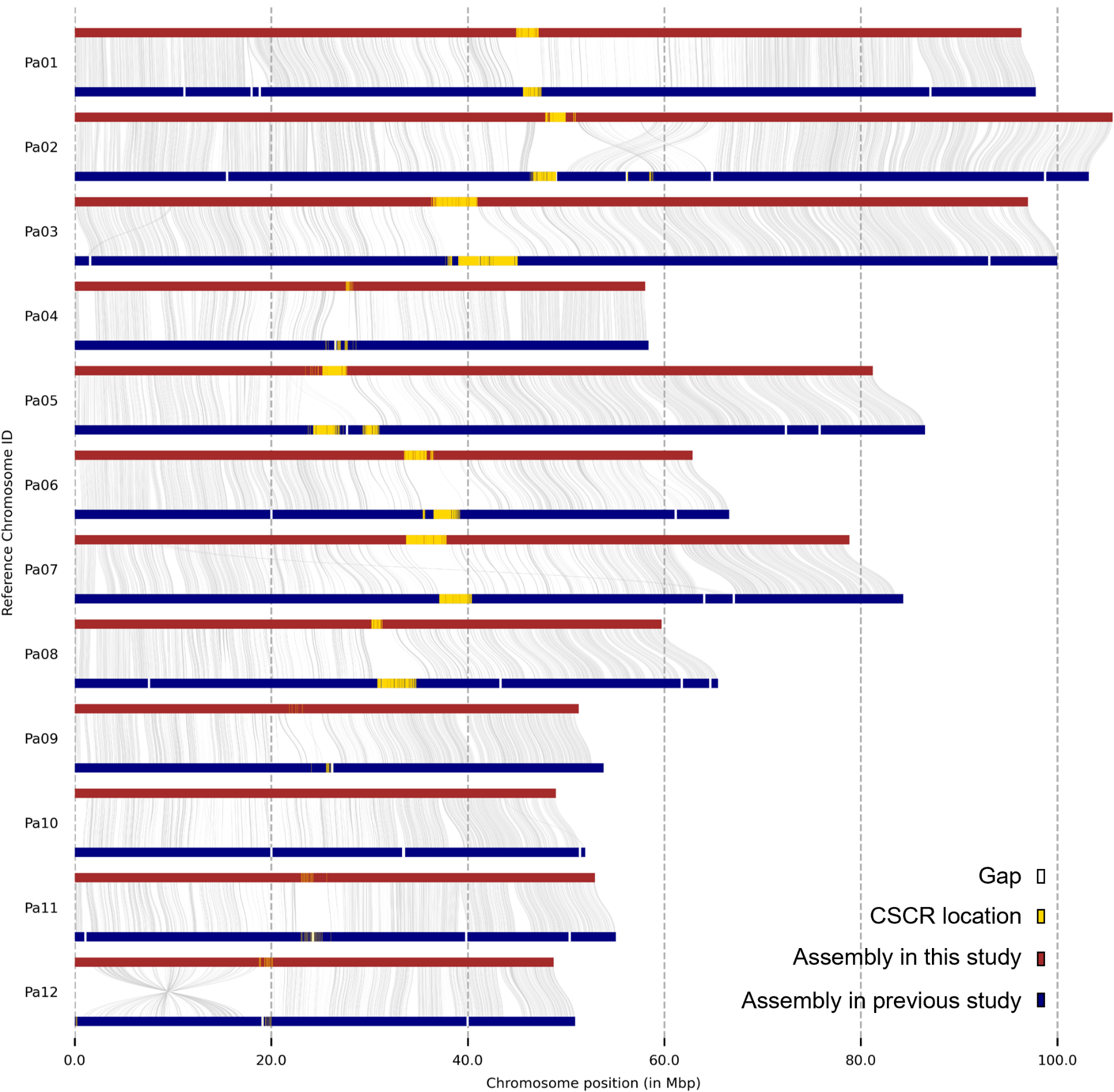


Figure S5 Gene-based colinearity between two avocado assemblies. The deep red chromosome schematic diagrams represent the chromosomes generated in this study and the dark blue ones represent the chromosomes generated in previous study. Homologous gene pairs identified by JCVI between two avocado assemblies are linked by gray lines at exact locations on correspoding chromosomes. Localizations of chromosome-specific centromeric repeats determined by LASTZ and gaps are marked in yellow and white respectively on corresponding chromosomes.


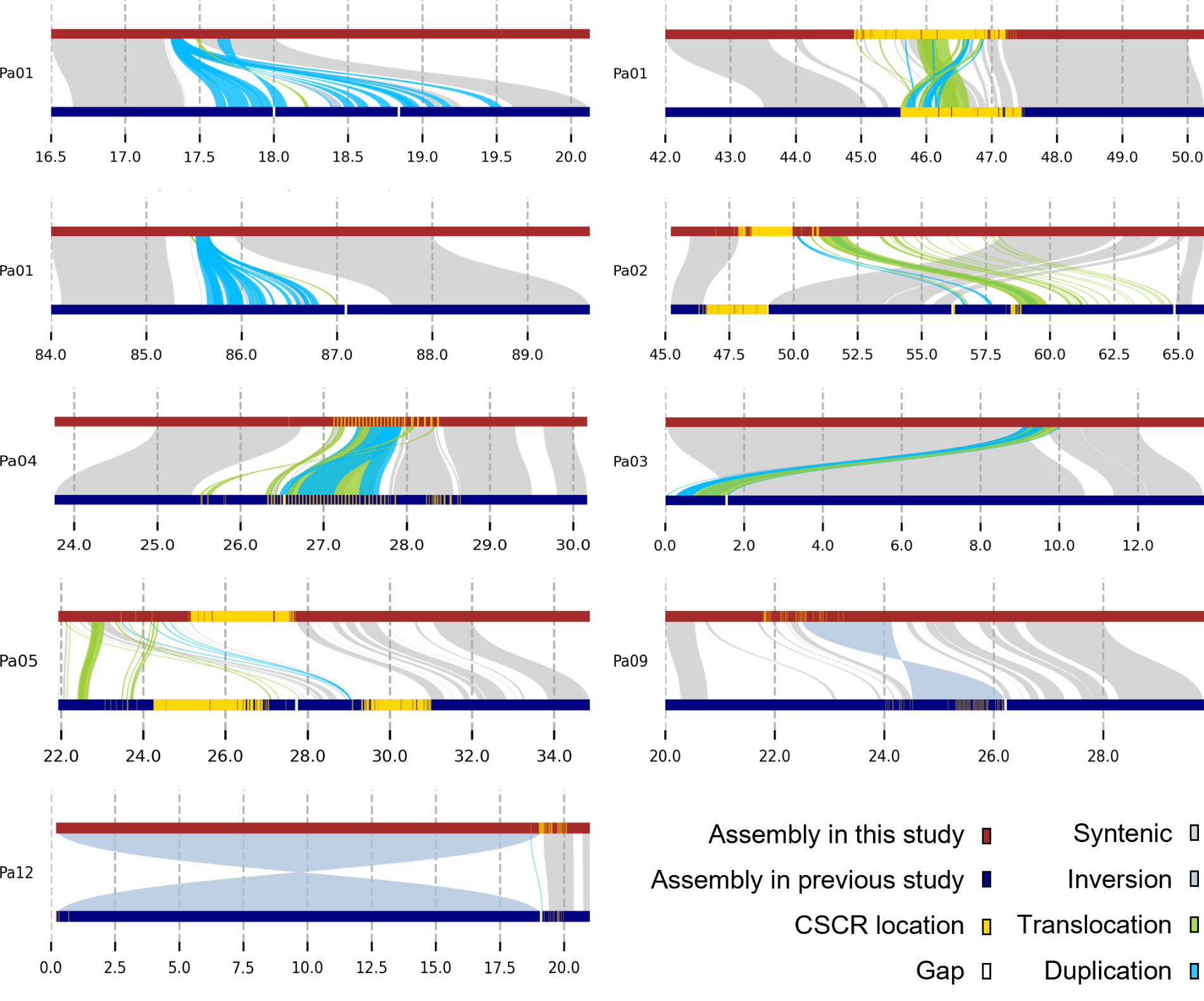
Figure S6 Micro collinearity diagrams showing the large-scale structural rearrangements between two avocado assemblies. The deep red chromosome schematic diagrams represent the chromosomes generated in this study and the dark blue ones represent the chromosomes generated in previous study. Syntenic regions, inversions, translocations, and duplications are marked between chromosomes. Localizations of chromosome-specific centromeric repeats determined by LASTZ and gaps are marked on corresponding chromosomes.

Figure S7 Localizations of *NLR* genes and their homologies between two avocado assemblies. The pink chromosome schematic diagrams represent the chromosomes generated in this study and the blue ones represent the chromosomes generated in previous study.
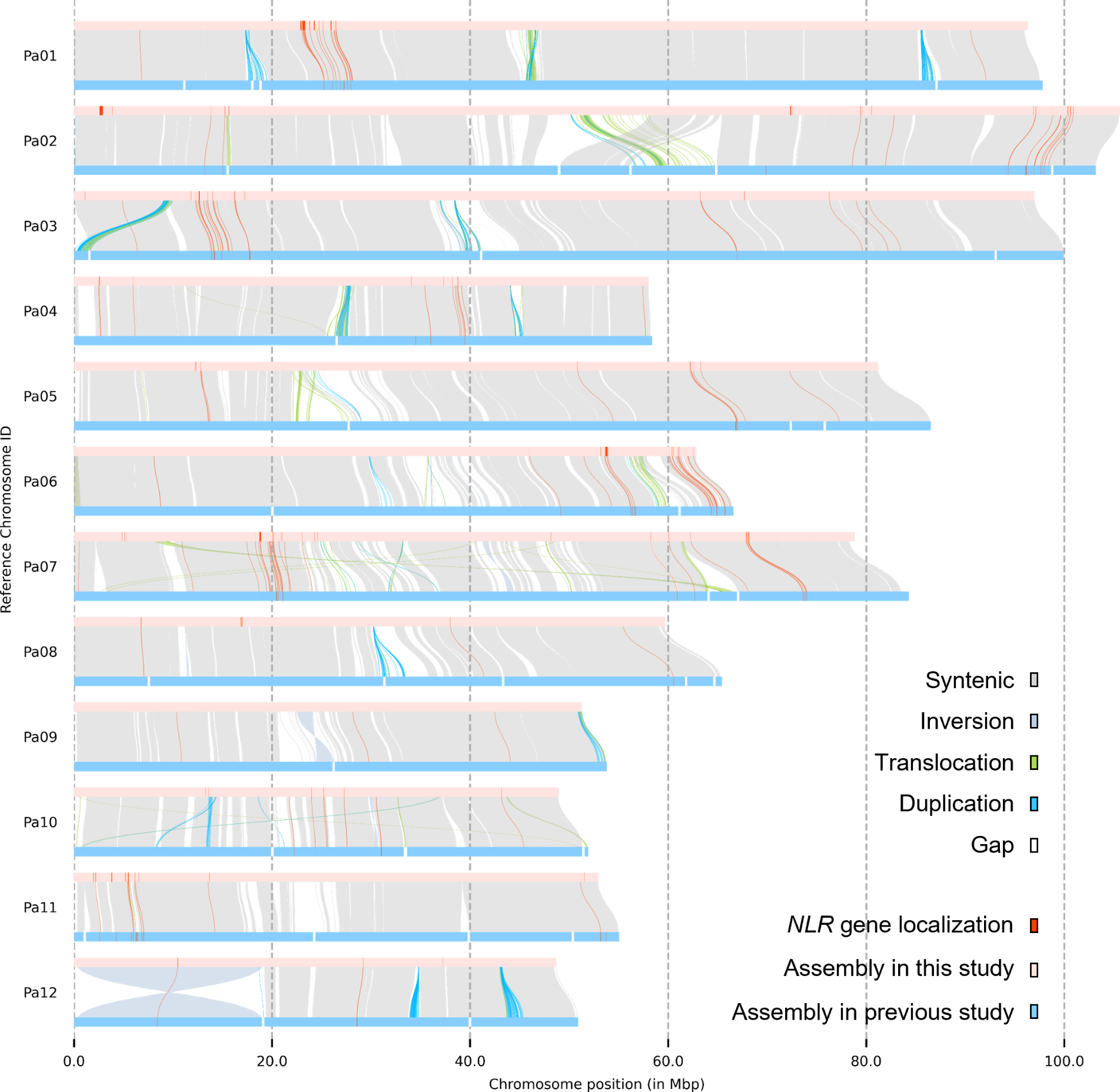
 Homologous *NLR* gene pairs are linked by red lines.


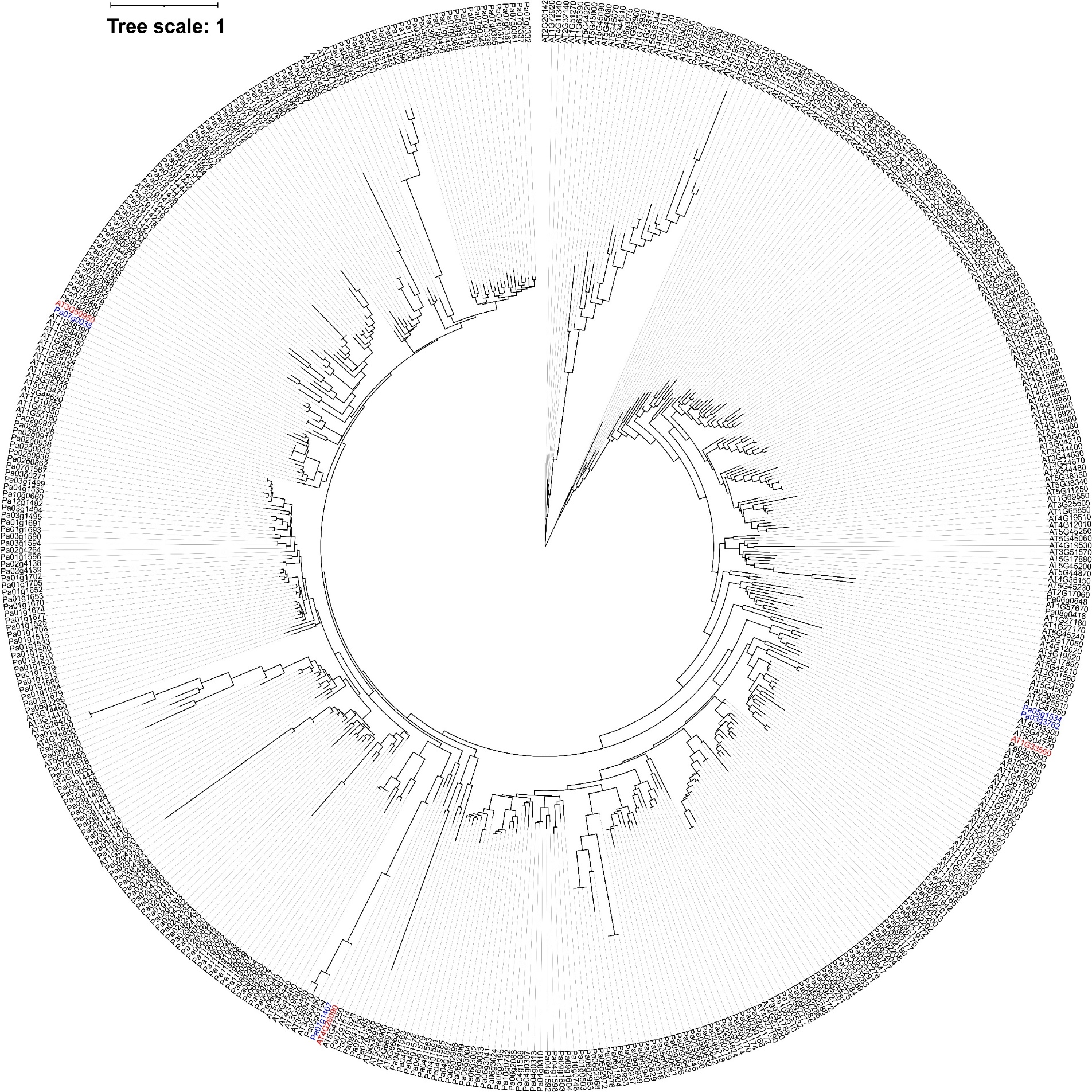
Figure S8 Phylogenetic relationships among the NLR proteins of *Arabidopsis* and avocado. The *NLR* genes in *Arabidopsis* were identified from Araport11 annotation set using the same method as avocado. These proteins were aligned by MAFFT einsi algorithm and trimmed by trimAl. The Neighbor-joining tree was constructed by TreeBeST (nj -t kimura -W -b 1000). Red labels indicate the functionally validated disease resistance genes in *Arabidopsis*. Blue labels indicate the homologous genes in avocado.
